# “The whole world is becoming more like Russia.” A conversation on deglobalization in the wake of the war in Ukraine

**DOI:** 10.1007/s11609-022-00482-w

**Published:** 2022-09-19

**Authors:** Boris Kagarlitsky, Janina Puder, Stefan Schmalz

**Affiliations:** 1 Moscow School of Social and Economic Sciences, Gazetny Pereulok, 3–5, 125009 Moscow, Russian Federation; 2 Institute of Sociology, Friedrich-Schiller-University Jena, Carl-Zeiß-Str. 3, 07743 Jena, Germany; 3 Faculty of Economics, Law and Social Sciences, Erfurt University (Campus), Nordhäuser Str. 63, 99089 Erfurt, Germany

**Keywords:** Sanctions, Gas, China, Socio-ecological transformation, Capitalism, Deglobalization, Sanktionen, Erdgas, China, Sozial-ökologische Transformation, Kapitalismus, Deglobalisierung, Sanctions, Gaz, Chine, Transformation Socio-écologique, Capitalisme, Démondialisation

## Abstract

The Russian invasion of Ukraine is having profound repercussions for the international system and the global economy. In this conversation, Boris Kagarlitsky, professor at the Moscow School for Social and Economic Sciences and long-standing analyst of Russian society, politics, and the global political economy, discusses the implications of the war on the Russian economy, its financial sector, and the Russian elite. Furthermore, Kagarlitsky analyzes the ongoing crisis of globalization, in particular Western sanctions, rising commodity prices, and the current role of China.

## Abstract (de)

Die russische Invasion in der Ukraine hat tiefgreifende Auswirkungen auf das internationale System und die Weltwirtschaft. In diesem Gespräch erörtert Boris Kagarlitsky, Professor an der Moskauer Schule für Sozial- und Wirtschaftswissenschaften und langjähriger Beobachter der russischen Gesellschaft, Politik und der globalen politischen Ökonomie, die Folgen des Krieges für die russische Wirtschaft, den Finanzsektor des Landes und die russische Elite. Darüber hinaus analysiert Kagarlitsky die gegenwärtige Krise der Globalisierung, insbesondere die Sanktionen des Westens, steigende Ressourcenpreise und die aktuelle Rolle Chinas.

## Résumé

L’invasion russe en Ukraine a eu des répercussions énormes sur le système international et l’économie mondiale. Dans cette conversation Boris Kagarlitsky, professeur à l’École des sciences sociales et économiques de Moscou et analyste de longue date de la société russe, de la politique et de l’économie politique mondiale, aborde des implications de la guerre sur l’économie russe, son secteur financier et l’élite russe. En outre, Kagarlitsky analyse la crise actuelle de la mondialisation, notamment les sanctions occidentales, la hausse des prix des matières premières et le rôle actuel de la Chine.

The Russian invasion of Ukraine has not only caused death, destruction, and dislocation, but has also had a huge impact on the international system and the world economy. Western sanctions have even exacerbated the already visible war-related disruptions to trade and finance. Several packages of sanctions imposed by the U.S., EU, Great Britain, and their allies have decoupled important parts of the Russian economy, the eleventh largest economy worldwide in 2021, from global markets. Simultaneously, EU member states have questioned their high dependency on Russian oil and gas imports and their geo-economic approach of “change through trade” (“Wandel durch Handel”, a truism among Germany’s political elite). Many observers, such as NY Times staff editor Spencer Bokat Lindell (2022), have even questioned whether the war in Ukraine might “spell the end of globalization” as the world experiences a period of economic deglobalization (see also Posen 2022).

In the wake of these developments, it is uncertain how long the war in Ukraine will last and which direction it will take. For the global economic and political system, however, it could become—much like the Covid-19 pandemic—a historic “point of bifurcation” (Rosa 2020). Its impact could be far-reaching and trigger societal changes: *First*, the conflict is creating new fault lines and fractures in globalization. Against the backdrop of looming gas shortages and supply chain disruptions, major transnational corporations and Western governments are reassessing the geopolitical risk of globalized production. “Friendshoring” (Maihold 2022) is the new buzzword of leading think tanks, which argue that geopolitical risks must be taken into account when establishing global supply chains. *Second*, as Putin’s attempt to annex Ukraine in a brief war is faltering and Western sanctions are hitting the Russian economy, the Russian government is forced to forge new partnerships. One of Russia’s closest allies at present is China, a huge potential market for Russian oil and gas exports. In addition, China would be able to support the troubled Russian economy with much-needed technological products and spare parts but has so far been reluctant to risk Western sanctions against Chinese companies. *Third*, the Ukraine war took the EU by surprise, as it is undergoing a far-reaching process of socio-ecological transformation and seeks to achieve carbon neutrality by 2050. This “new great transformation” (Dörre et al. 2019), driven by the “European Green Deal”, is currently under great pressure as Russian gas and oil imports need to be quickly replaced by other energy sources. This transition has led to a boom in (even dirtier) liquefied natural gas imports from the US and to a potential revival of nuclear energy. *Fourth*, rising living costs due to higher oil, gas, and food prices will have a global impact. A new inflation-driven “cycle of contention” (Tarrow 1998), accompanied by strikes and social protests in Europe and food riots in Africa, is a possible scenario for late 2022. While the political fallout of such a “winter of discontent” is hard to predict, protests are likely to influence politics in many gas and food-importing countries. However the global economy evolves, the war will have a lasting impact on the socioeconomic status and life chances of large segments of the world’s population. *Fifth*, it is difficult to analyze the global impact of the war in Ukraine without situating it within the long durée of the global financial crisis of 2008/09. Several scholars have argued that neoliberalism as well as the Russian oil-dependent development model have been in crisis ever since (Johnson and Köstem 2016). Today, the war and sanctions have forced many Western companies to leave Russia and the Putin regime to embark on a shaky path of a more inward-looking economic model with unclear long-term outcomes. The Ukraine war therefore raises many questions about the future of Russia and the capitalist world-system. To answer some of these questions, we conducted an interview with Boris Kagarlitsky, a professor at the Moscow School for Social and Economic Sciences and a long-standing analyst of Russian society, politics, and the global political economy. In the interview, Kagarlitsky, author of books such as *The disintegration of the monolith* (Kagarlitsky 1993) and *Empire of the periphery. Russia and the world-system *(Kagarlitsky 2008), analyzes the current state of the Russian society, economy, and the global impact of the war.

## StS (Stefan Schmalz):

Following the Russian invasion of Ukraine, the US and its allies have agreed on far-reaching sanctions against Russia. Can you tell us more about the larger political and economic context of these sanctions?

## BK (Boris Kagarlitsky):

Ironically, the current geopolitical conflict and sanctions are not the cause, but rather the consequences of a broader process that already began with the Great Recession of 2008–2010. It became obvious that not only globalization and neoliberalism had reached their limits, but all over the world, in the West, in China, in Russia, and in Ukraine, a transition to a new development model became inevitable, yet clashed with the political and social status quo. This constellation has led to the present catastrophe. The current situation is therefore an example of the “dialectics of history”: When there is a contradiction between the objective needs and logic of the process and the subjective interests of the groups in power, catastrophic events, major wars, revolutions, global crises, and so on occur. In the case of Russia, its neoliberal development model was already under distress since the Great Recession of 2008–2010. Today, there is major social tension in Russia. Since the pension reform in 2018, the Russian regime has become increasingly unpopular. Russian elites tried to repeat history. When the Crimean Peninsula was taken from Ukraine in 2014, there was a wave of patriotism. But today the situation is different, and Putin and his entourage completely miscalculated the course of the war. Now the Russian elites are trying to blame everything on the West and Ukraine, when in fact the war was a consequence of their own mismanagement.

## StS:

Let us take a closer look at the sanctions imposed. One particular measure was targeted at the financial sector. For instance, important Russian financial institutions were excluded from SWIFT and transactions by the National Central Bank of Russia were prohibited. Can you explain the impact of these sanctions on the Russian banking system, the economy, and the population more generally?

## BK:

I think at this point the Russian banking system is extremely stressed, but so far, it has not collapsed, and I do not think it will, due to the nature of the Russian banking system, which is closely linked to the state and major corporations. It is not just about the financial system, but also about oligarchic rule. However, banks and financial institutions are causing great stress in the economy and society. While everything continues to function, it does so poorly. Ordinary people face many technical problems in their daily life, for example, when sending or receiving money from abroad or even when transferring money to another Russian bank account domestically. These problems are often minor, but nonetheless time-consuming. For instance, I can no longer transfer money using my cell phone like I used to. Moreover, international transfers are becoming more expensive. Financial flows are now running through financial centers in Ukraine, Kazakhstan, and Uzbekistan, which make a huge profit by enabling Russian international transactions. But this method only works for individuals and small and medium-sized enterprises. When millions of dollars are at stake, it will not work this way. For large companies, the situation is far worse, particularly in the manufacturing sector. Without the international payment system, Russian manufacturers cannot sell their products abroad nor finance and pay for imported goods. In addition to financial sanctions, the Western economic sanctions impede access to important components and spare parts. For instance, Russian car manufacturers are currently in serious trouble.

## StS:

Recently it was reported that in the wake of the sanctions the Russian automotive industry is now forced to rely on Soviet models due to a lack of spare parts. Can you tell us more about the impact of the sanctions on the manufacturing sector?

## BK:

For the first two or three months, the sanctions had no significant impact on the Russian manufacturing sector. Ironically, the low efficiency of the Russian industry became a favorable factor in withstanding the sanctions. Many Russian companies are accustomed to accumulate large inventories of equipment and spare parts. This is a tradition tracing back to Soviet times, due to a lack of trust most companies have in their suppliers and logistics networks. Curiously, this habit became a kind of remedy during the sanctions, in particular during the first two or three months. This was one of the reasons why Russian politicians at first triumphantly declared that Russia was not even feeling the sanctions, which was true to some extent. But this situation did not last very long. Around June, the Russian industry started running out of stocks. And since July, it has become possible to observe this economic decomposition, which is currently underway, in real time.

## StS:

Can you give another example of the economic decay in Russia?

## BK:

Yes, let’s take the transport sector, for example. Today, Aeroflot is cannibalizing its own aircraft. It is not able to use all its aircrafts because many international flights have been cancelled. So, Aeroflot is disassembling its aircrafts to obtain spare parts for repairs. However, smaller provincial airline companies with approximately ten airplanes cannot do this, and so quite a few of them are going out of business. For some regions in the far east and the far north this is very tragic. They are unable to sustain communication with the rest of Russia and with the rest of the world. Hence, it is becoming a serious problem in places like Yakutia, where I was in February. By the way, this is one of the few regions that retains some elements of democracy and political freedom. There have been no political arrests in this region. However, I spoke to local representatives of the regional Republican Assembly back in February, and they were already extremely concerned that Yakutia will be cut off from the rest of the country, especially from air transport.

## JP (Janina Puder):

As you explained so far, the sanctions entail comprehensive consequences. The Russian Railways even requested the EU to lift the sanctions imposed on it, as they negatively impact ordinary citizens.

## BK:

Yes**, **they declared it publicly. But I seriously doubt that the West is going to rescue Russian Railways, because it is also used to transport military supplies. From a military perspective, it is even more important than civilian air transport, which is a far more expensive way to move troops from one part of the country to another. The problem is that locomotives, like most branches of the Russian industry, depend on foreign spare parts and technology. And with the policy of so-called “import substitution”, which started with the sanctions against Russia over the takeover of Crimea in 2014, this Russian dependency has even increased. At that point, Russia started to produce products labelled “Made in Russia” by assembling foreign spare parts in order to receive subsidies.

## StS:

From a historical perspective, import substitution was considered an important strategy of late industrialization. For instance, after the Great Depression of 1929, international trade collapsed and as a result of the breakdown, many Latin American countries pursued a domestic market-driven industrialization strategy. Several scholars from the dependency school, such as Raúl Prebisch (1950) and André Gunder Frank (1966), later argued that the Great Depression was paradoxically a window of opportunity for national development. What about Russia today, has import substitution also had a positive impact?

## BK:

I think that import substitution simply will not work in a neoliberal market economy and with a corrupt government. It is not going to work without a “strong state” and capital controls. So far, Russian import substitution has been mainly about receiving subsidies or benefits from government programs, and for this reason producers just had to label their products with “Made in Russia”. The easiest way to do this is to buy foreign parts and assemble them in Russia. Russian import substitution was often about replacing Western imports by Chinese ones. In some cases, this even included fake Russian products. There are jokes about Belarussian prawns. But Belarus has no sea; nevertheless, the country managed to supply Russia with a lot of seafood. Why? Because Belarussians bought products from Poland or from Norway, repackaged them, and labeled them “Made in Belarus” and resold them to Russia.

## JP:

Do you think that the Russian style “rentier capitalism” (Christophers 2022), based on the ownership of natural resources of a small oligarchy, impedes import substitution?

## BK:

Yes, exactly! Import substitution can work, but only if the government and the state are not dominated by a neoliberal oligarchy and the export of raw materials. And even if the Russian government were to seriously try now, it would be too late. Today, the government often refers to the Soviet experience of the 1930s, but there is a huge difference: In the 1930s, the Soviet economy was not isolated from the rest of the world, it was not subject to sanctions. On the contrary, it was the Soviet Union’s honeymoon of international trade, because during the Great Depression the whole world was happy to sell their products to the Soviet Union to compensate for the loss of export markets elsewhere. Hence, the Soviet Union benefitted from the massive sale of cheap technology and equipment. Consequently, Soviet industrialization was not only based on collectivization with its many victims, but also on international trade. I have described this process in my book *Empire of the Periphery* (Kagarlitsky 2008; see also Shubin 2009; Kolganov 2018). Today, Russia’s elite fails to solve the problems of import substitution and re-industrialization for similar reasons as a decade ago, but the situation is getting much worse. The government faces huge financial problems.

## JP:

What kind of financial problems?

## BK:

There is a strange side-effect of the sanctions. The ruble became stronger, although neither the West nor the Russian government wanted to strengthen the ruble. Everybody expected the ruble to weaken because of the sanctions. The West did not want to strengthen the ruble, on the contrary it wanted to weaken the Russian financial system. But the interesting aspect of this story is the reason why the Russian government is seriously concerned about the strong ruble. After all, the Russian revenues are based on export earnings from oil and gas and a few other raw materials. For this reason, a weak ruble is extremely helpful to finance the Russian state. Today, the same export earnings will result in about 30 % less expenditures on pensions, salaries of state employees, social benefits, roads, and money for the state bureaucrats themselves. Once again corruption plays a role: In Russia today, at least 20 % or even 30 % of the funds for important projects are stolen. And the lack of funds is also affecting Russian war efforts.

## StS:

And why did the ruble appreciate that strongly?

## BK:

It is quite unusual, but it is a logical consequence of the sanctions. International trade has collapsed, tourism has collapsed, and when you cannot buy anything from abroad, you are not interested in purchasing foreign currency. You simply do not need it. As imports have collapsed, so has the demand for foreign currency. Large parts of the Russian middle class have accumulated their savings in U.S. dollar or Euros. However, their savings have lost value instead of guaranteeing financial protection in times of crisis. Quite a few of them tend to believe in a conspiracy of the central bank or the government stealing their savings. At the same time, prices are rising, and people only have little money for their daily lives as incomes are shrinking. As a result, there is low trust in the ruble even though it is appreciating. Many people and companies are short of money. However, the central bank and the Minister of Finance dread inflation. That is why they refrain from printing money, so they do not fill the gap with liquidity. This is a very interesting phenomenon in economic history, it is a strange combination of deflation and stagflation at the same time. It is like a bizarre meeting of the German Reichskanzler Heinrich Brüning (1930–1932) and the British Chancellor of Exchequer Iain Macleod (1970). I think the situation in Russia is unique in many ways. I have never encountered anything like it. So, I think the financial sanctions are working, but not as originally planned.

## StS:

Many Western companies have announced their withdrawal from the Russian market, for instance McDonalds has left Russia. It was bought by the Siberian oligarch Alexander Govor and was rebranded as “Vkusno i tochka” (“Tasty and that’s it”). How is the domestic market in Russia changing?

## BK:

Well, things are getting worse, but not right away. And, of course, many jobs will get cut. In the case of fast-food chains like McDonald’s, this is something that can be replaced. They can produce hamburgers, even with little technology, especially because most of the equipment was left behind when McDonald’s withdrew from Russia. Some people complain about the quality. But it depends on the sector, as I already elaborated regarding the manufacturing sector. And in some cases, when foreign companies leave, some Russian competitors will even benefit from it. So, there are a few sectors of the Russian economy where there will be local winners of the crisis. This has always been the case. Among small and medium-sized enterprises, the picture is mixed. You hear terrible stories about people who have been ruined. But there are also a few success stories of Russian companies that are taking the opportunity to fill the emerging gaps left by the departure of foreign competitors. One could say: Nothing fails like failure, and nothing succeeds like success. But in general, the economic situation is deteriorating.

## StS:

I think there is an elephant in the room: Another option to fill the emerging gaps would be China. Russia and China have developed very close ties in recent years. The big question is whether Chinese companies will step in. Will Russia “reOrient” (Frank 1998), as some scholars suggest (e.g. Lukin 2018)?

## BK:

No, I do not think so. Russia is already selling everything it can sell. And Chinese interest in Russia is decreasing. The Chinese government and companies are avoiding risks as Russia is potentially becoming a very unstable and dangerous place. They are concerned about the business environment. The Russian market is shrinking. Of course, the Russian government expected the Chinese to replace the Westerners, but in fact they are leaving the country along with the Westerners. So Russia’s journey eastwards, as it is called in Russia, is not working.

## JP:

Because Chinese companies want to avoid potential sanctions?

## BK:

Yes, there is a risk of sanctions. Chinese companies might increase their market shares in some products like refrigerators, cell phones and a few other things. But when it comes to strategic products such as microchips, Chinese companies will not help. And as I said, Chinese businessmen are cautious, they tend to avoid unnecessary risks. Things will probably change once Putin goes, but so far we do not know when and how he will leave. So Chinese companies are waiting, they will still have the time and the opportunity to fill the gaps left by Western companies.

## StS:

And what about the Russian oligarchs and state officials who have also been sanctioned. Are they still close to the Putin regime or what is their stance now?

## BK:

When the war started, we had a very interesting discussion with a few critical intellectuals here about the capitalist interest in the war. And everybody said that the dominant oligarchic interest groups in Russia are losing nowadays. Their situation is worsening. Their only interest was not to lose the war. That does not mean that they did not have any interest in the war, they simply expected the war to last for one or maybe two weeks. This was the worst-case scenario they envisioned. And within that scenario, there would have been plenty of opportunities to loot Ukraine. But this did not happen. And now they are on the losing side. As I said, the situation started to deteriorate long before the war. I think the war itself is a consequence of the crisis rather than the cause of the crisis. And in that sense, the war seemed to be a kind of magic bullet. But instead of benefitting the oligarchs, the war made their problems much worse. And as far as I know, these people are extremely depressed at this point. They have increasing doubts about Putin and his closest entourage, and about their own future with Putin.

## JP:

Given the skepticism of the “old” ruling class towards Putin and the problems they now face due to the economic consequences of the war, are there new conflicts emerging among the Russian ruling class? And is there a fraction gaining from the war?

## BK:

There are two important developments: First of all, the regime itself is becoming increasingly authoritarian, centralized and irrational. The circle of those who have access to the decision-making process is getting narrower and narrower. Many individuals, corporations, and interest groups that used to be extremely influential are now completely cut off from the decision-making process. And another interesting phenomenon that I describe in my new book, which I am currently writing, is that the centralization of the Russian state is a by-product of its low efficiency, because the central government does not trust the local bureaucracy and even the middle level bureaucracy, partly because of the low quality of their management. This leads to a vicious circle. The central state deprives them of capacities, resources, and decision-making power, which leads to a further deterioration in their quality and the need to recentralize power. This process has been going on for years and has led to an absurd situation due to the grotesque level of centralization. Today, many decisions are made by Putin alone, with very little consultation, which has led to certain degree of discontent among important sectors of the bureaucracy. And at this point, I do not see any way to solve this problem unless Putin dies or there is a coup d’état. There is a growing estrangement between Putin and his closest friends and the rest of the Russian ruling class. There are emerging conflicts within the ruling class because they do not have any clear strategy. They are beginning to fight each other. Second, there is also a new fraction emerging within the elite, which is benefiting from the war. This group is satisfied with the current situation and does not belong to the military. These are people from the propaganda sector, which has developed into a major industry with enormous investments that have grown even more during the war. The worse the situation is on the front and the worse the situation is internationally, the more money flows into propaganda. Another sector is made up by the security apparatuses. Not the military, though, which is humiliated by the current course of the war. But the police, the FSB (Federal Security Service of the Russian Federation), the secret security services and the propaganda sector are becoming a new base for the regime at this point.

## JP:

So the old elite is becoming more fragmented and new rising ruling class fractions are more authoritarian minded?

## BK:

That is absolutely correct. The problem is that the new ruling class is formed by people who are not producing anything in a capitalist sense. They are not generating any profits, even the traditional oligarchs managed to do that. They merely squeeze resources out of the economy. And this huge apparatus of squeezing resources out of the economy is not only squeezing resources out of ordinary people, but also out of the oligarchs. So, this is where new conflict lines emerge within the Russian elite.

## JP:

I would like to shift the perspective. The sanctions imposed on Russia seem to be backfiring on Western countries. For instance, inflation is currently rising in the EU, and oil and gas prices on global markets have increased tremendously. Against the backdrop of the profound socio-ecological transformation in Western societies (see Dörre et al. 2019), these developments are likely to result in social conflicts. What is the impact of the war on Western economies and societies?

## BK:

Let’s start with the fact that, on the one hand, global structural change and economic restructuring were already taking place before the war. This can be seen, for example, in the EU’s ambitious plans for technological innovation and energy transition to become less dependent on fossil fuels. In a certain sense, the war is accelerating this process. On the other hand, the question arises as to who is going to pay for this massive transition. This question generated contradictions within Western societies. A good example is the French Yellow Vest movement, Gilets Jaunes. The movement refused to accept that ordinary people should pay for environmentalist policies, the so-called Carbon Tax, and argued that these policies are designed to benefit large corporations. It is interesting, then, that the progressive demand for environmental policies was appropriated by the elites in most Western countries and transformed into an effective tool to increase the exploitation of ordinary people. The war is a game-changer: Part of the conflict over rising prices and the costs of energy transition will be externalized. The war is a very useful scenario for Western elites. In the context of the ongoing structural transition, the war against Putin is a stronger justification than the need to save the planet. And Russia will pay a high price.

## JP:

However, most economies are currently still heavily dependent on fossil fuels. Is the global race for fossil raw materials intensifying as a result of the supply bottlenecks that have arisen in the wake of the Ukraine conflict, in which gas supplies are also being used as a leverage against the West?

## BK:

Currently, the war is speeding up the process in which Western countries seek to substitute oil with other resources. But it has accelerated this process to the extent that the West will not be able to replace it with renewable or other non-renewable resources in the short run. The solution seems to lie in a redistribution of the oil market. Some of Russia’s former market shares might go to Saudi Arabia and some even to Venezuela or Iran. In addition, many states will also have to consider returning to nuclear energy. In terms of emissions and climate impact, nuclear energy appears less problematic in the short term than fossil or agrofuels. Ironically, it also appears to be the safest option. Either way, Russia currently appears unwilling to support the West in carrying out its original transformation agenda.

## JP:

What is Russia’s strategy in the global competition for resources given its role as a gas supplier?

## BK:

This is very tricky, because on the one hand Russia needs to sell its gas, as it is an important and stable source of revenue. On the other hand, Russia uses gas as a weapon to put pressure on its adversaries or partners. However, this tactic paralyses its own decision-making because both tendencies contradict each other. In the short run, Russia will try to balance these tendencies by cutting sales and raising prices. This could lead to constant shockwaves. Russia will probably oscillate in its tactics. So I do not think Russia will let Europe freeze, but it will swing back and forth with its gas supplies making everything less stable and predictable. This could lead to an accumulation of stocks in the West in case Russia eventually cuts its gas exports. In that sense, the whole world is becoming more like Russia.

## StS:

The current crisis, the Ukraine war and the rising oil prices, resembles the triggers of the end of the “golden age of capitalism” (Marglin & Schor 1990) in the 1970s in the Western world. The Vietnam War and the oil price shocks of 1973 and 1979 put massive pressure on the global financial system.

## BK:

Well, both the Vietnam War and the Six-Day War between Israel and Egypt, Syria and Jordan led to far-reaching transformations in the oil market. The latter had an even greater impact than the Vietnam War. By the mid-1970s, both the Keynesian model in the West and the Soviet model had been exhausted. The wars and conflicts during this period resulted in a new dynamic which led to the rise of neoliberalism in the early 1980s. I am not saying we will now return to Keynesianism, but today it is clear that the Polanyian pendulum (Polanyi 2001) will swing in the other direction. Of course, there are social forces which oppose this change. Neoliberalism was the triumphant period for the political right and the capitalist class. Perhaps the left and the working class will get their revenge in the long run.

## JP:

So how can the current crisis be understood from a theoretical perspective?

## BK:

My personal approach is to analyze the history of capitalism. And as Immanuel Wallerstein (2004) and also the Russian historian Mikhail Pokrovsky (1925) point out, capitalist economies have developed in cycles. There have always been periods of globalization and deglobalization. The periods of globalization have usually been dominated by trade and financial capital. Without using the terms globalization or deglobalization, which was later developed by the Philippine sociologist Walden Bello (2002), Pokrovsky and also Weber (1981) argued that nation-states tend to compete for mobile capital, while industrial capital is fixed and cannot move as quickly. The history of capitalism is thus a history of cycles of globalization and deglobalization, with the alternation of cycles usually also accompanied by major social conflicts and wars. For instance, according to Immanuel Wallerstein, the long-sixteenth century was a period of globalization while in the early 17^th^ century mercantilist Colbert-style economies prevailed in most European countries such as France or Russia.

## JP:

If we look 10 to 15 years into the future, what could a globalized or maybe deglobalized world look like?

## BK:

We will definitely experience some form of deglobalization. The role of nation-states will increase, but they will transform dramatically—maybe to the extent that they will no longer be recognizable as nation-states in the current sense. New globalized political formations may emerge that reach beyond the nation-state and are not based on open market policies. What we would actually need is some form of democratic planning and coordination and a new form of welfare state that goes beyond the traditional bureaucratic model. Therefore, we must rediscover democracy. Christopher Lasch (1996) and others have pointed to the fact that many formal democracies have been hollowed out by the elites over the last 20 to 30 years. Rediscovering democracy would include a form of participatory democracy from below, including economic democracy. In my opinion, the European Union in its current form has no future. But maybe a new Europe could emerge from the current crisis. At the moment, we are discussing Ukraine’s possible membership in the EU. Perhaps some kind of new Congress of Vienna is needed, bringing together all of Europe, including Ukraine and Russia, in order to conclude a new treaty for Europe. Instead of a neoliberal treaty, like the Treaty of Maastricht or Lisbon, such an arrangement would prioritize people’s rights over bureaucracies. But everything is open for struggles. It is not about predicting the future, but about fighting for it.
